# Gene expression profiling in pbMEC – in search of molecular biomarkers to predict immunoglobulin production in bovine milk

**DOI:** 10.1186/s12917-017-1293-z

**Published:** 2017-11-29

**Authors:** M. Hillreiner, C. Schmautz, I. Ballweg, V. Korenkova, M. W. Pfaffl, H. Kliem

**Affiliations:** 1 Chair of Animal Physiology and Immunology, Technische Universität München, Weihenstephaner Berg 3, 85354 Freising, Germany; 2 Quantitative and Digital PCR Core Facility, Institute of Biotechnology CAS, v. v. i. BIOCEV Center, Vestec, 252 50 Prague, Czech Republic

**Keywords:** Molecular biomarkers, Primary bovine mammary epithelial cells, Innate immunity, Microfluidic gene expression profiling, *Clostridium difficile*-associated diarrhea

## Abstract

**Background:**

Optimization of the immunoglobulin (Ig) yield in bovine milk used as therapeutic immune milk or whey for the prevention of *Clostridium difficile*-associated diarrhea in humans is of great importance to improve the economic efficiency of production. Individual dairy cows have diverse immune responses upon vaccination, resulting in a variable Ig yield in blood and milk. Therefore, it is advisable to pre-select cows with the best ability to produce and secrete high yields of specific Igs.

**Results:**

The gene expression profile of pbMEC (primary bovine mammary epithelial cells), challenged with the gram-positive, non-mastitis, pathogen *Clostridium difficile* showed distinct and significant differences in the gene expression of effector molecules of the innate immune system. A number of genes were identified that could possibly serve as molecular biomarkers to differentiate high responder cows from low responder cows. These identified genes play key roles in the promotion of innate immunity.

**Conclusion:**

Using a gene expression profiling approach, we showed that upon others, especially the gene expression of the pro-inflammatory cytokines was altered between the high and low responder cows. Those genes are indicated as potential molecular biomarkers in the pre-selection of cows that are able to secrete high immunoglobulin yields in milk.

**Electronic supplementary material:**

The online version of this article (10.1186/s12917-017-1293-z) contains supplementary material, which is available to authorized users.

## Background

*Clostridium difficile* (*C. diff.*) is a widespread hospital germ that causes severe antibiotic associated gastroenteritis in humans especially in industrialized countries [1, 2]. *C. diff.* is a gram-positive enterotoxic, spore building pathogen that due to its acidic resistance is able to overcome the acidic environment of the stomach [1–3]. The primary reservoirs of this pathogen are asymptomatic carriers and contaminated surfaces, which are important issues especially in hospitals and nursing homes [4]. The progression of the disease is quite diverse, ranging from mild diarrhea to severe life-threatening pseudomembranous colitis [1, 3, 5]. Until now, the treatment of *C*. *difficile*-associated diarrhea (CDAD) results in a vicious circle, as the antibiotics metronidazole, vancomycin, and fidaxomicin are mainly used [6–8]. As those antibiotics do not exclusively target pathogenic *C. diff.* Bacteria but also commensal gut bacteria, the gut microbiota of the patients is further damaged. Therefore, we aimed to develop a new treatment strategy or better yet, a preventive treatment strategy for CDAD. Inspired by a study by Van Dissel et al. (2005) [9], we wanted to developed immune milk enriched with naturally derived polyclonal immunoglobulin A (IgA) against *C. diff.*. As the production and application of immune milk are quite promising, it is advantageous to optimize the yield of specific IgA against *C. diff.* in the milk. Therefore, molecular and biological methods were employed to identify potential molecular biomarkers for the pre-selection of high responder dairy cows, prior to immune milk production. Brown Swiss cows were immunized against *C. diff.* in order to induce milk production and secretion of specific IgA. As each animal has a fairly individual immune status and, hence, response due to the inherited genetic composition of the host [10], we investigated whether animals can be pre-selected to optimize production of specific Igs upon vaccination. Therefore, we searched for molecular markers of the innate immune system of the cows using a gene expression profiling method. As we surmised that besides blood lymphocytes, primary bovine mammary epithelial cells (pbMEC) are quite important for the promotion of innate immunity and subsequent activation of adaptive immunity and later on, transcytosis and secretion of immunoglobulins into milk, a newly developed three-dimensional 3D–cell culture system of pbMEC was used in this study [11, 12]. The elucidation of the underlying gene expression network may be important to identify differences in the innate immune system of low and high responder cows to facilitate the pre-selection of animals before use for immune milk production.

## Methods

### Immunization of the cows

The animal trial was approved by the government of Upper Bavaria (AZ. 55.2–1–54-2532.6-17-2012). The cows were bought at the cattle market for Brown Swiss cows of the Allgäuer Herdbuchgesellschaft (Cattle’s breeders association). Nine healthy Brown Swiss cows in their first lactation were immunized against *C. diff.* (IDT Biologika GmbH, Dessau-Rosslau, Germany) according to a strict scheme of 16 immunizations over a 31-week period*.* Before and 1 day after each vaccination, the health status of each animal was routinely monitored by a veterinarian. The milk of each udder quarter was tested for bacterial infection and contamination (Tiergesundheitsdienst Bayern e.V., Grub, Germany) before vaccination to detect the incidence of subclinical or clinical mastitis. Somatic cell counts and milk ingredients were analyzed weekly by a commercial facility (Milchprüfring Bayern e.V., Wolnzach, Germany). The average somatic cell count in milk during the vaccination period was 63.000 cells/ml ± 7075.63 cells/ml (*n* = 279). During the 31-week experimental period, two cows developed symptoms of subclinical mastitis and one was diagnosed with acute mastitis. No pbMEC were sampled from the diseased animals, as only those from healthy cows were used in the experiments. Furthermore, stool analysis of the animals prior to immunization showed that all were *C. diff.* Negative (Leiden University, Medical Center).

### IgA against Clostridium Difficile - ELISA

For the detection of *C. diff.* Specific IgA in cow milk, a sandwich ELISA was applied. In brief, each well of a 96-well plate (Maxisorp, Nunc®; Sigma-Aldrich Corporation, St. Louis, MO, USA) was coated with 2.0 × 10^8^ *C. diff.* Cells/ml, IDT Biologika GmbH) in coating buffer (50 mM NaHCO_3_, pH 9.6; Merck Chemicals GmbH, Darmstadt, Germany) and incubated for 2 h at 70 °C and then overnight at 4 °C. The coating was terminated by incubation with 200 μl blocking buffer in phosphate-buffered saline (PBS)-Tween 20 (PBST; 2% gelatin, Sigma-Aldrich Corporation) for 1 h at 37 °C. The ELISA plate was washed four times with PBST (1 g/l Tween 20; Merck Chemicals GmbH). A *C. diff.* Specific IgA standard was prepared in dilution buffer (0.2% gelatin, Sigma-Aldrich, in PBST, 62.5 ng/ml – 4*10^3^ ng/μl). The skim milk samples were diluted to 1:10 with dilution buffer. Standard dilutions, samples, and intra-assay controls were applied in duplicates to the pre-coated plate and incubated for 1.5 h at 37 °C. Afterwards, the ELISA plate was washed four times. Then, horseradish peroxidase (HRP)-conjugated sheep anti-bovine IgA (dilution, 1:70,000; Bethyl Laboratories, Inc., Montgomery, TX, USA) was added to each well and the plate was incubated for 1.5 h at 37 °C in the dark. Afterwards, the ELISA plate was washed four times and the HRP-conjugated substrate [13] was added to the wells to induce reaction with the substrate. After 40 min, the substrate reaction was stopped by the addition of 2 M H_2_SO_4_. Extinction was measured after 30 min at 450 nm using a microplate reader (Sunrise™, Tecan Group Ltd., Männedorf, Switzerland). The amount of *C. diff*. Specific IgA was determined based on a standard curve using Magellan™ V6.6 software (Tecan Group Ltd.).

### 3D cell culture of pbMEC

The pbMEC were isolated from fresh milk of nine healthy Brown Swiss cows in mid-lactation, as described by Sorg et al. (2013a) and Danowski et al. (2013) [14, 15]. In brief, fresh milk was defatted (10 min, 1850×*g*), and the resulting cell pellet was washed several times with 1× Hanks Balanced Salt solution (Sigma-Aldrich) supplemented with antibiotics and antimycotics [14, 15]. The remaining cell suspension was filtered twice (EASYstrainer™; 40 μm; 100 μm; Greiner Bio-One GmbH, Frickenhausen, Germany) to remove lipid droplets and cell aggregates. The pbMEC were afterwards resuspended in Dulbecco’s modified Eagle’s medium (DMEM)/F-12 Ham solution supplemented with penicillin/streptomycin, amphotericin B, insulin-transferrin-selenium (ITS) liquid media supplement (Sigma-Aldrich) and fetal bovine serum (FBS; Gibco® Lifetechnologies GmbH, Darmstadt, Germany), and cultured (37 °C, 5% CO_2_) in 3D cell culture in 6-well plates coated with 2.4 mg/ml Matrigel® (Corning Inc., Corning, New York, USA), until confluency. pbMEC were sub-cultivated using 0.25% Trypsin-EDTA solution (Sigma-Aldrich). After the second passage, the cells were detached with 0.25% Trypsin-EDTA solution and prepared for cryopreservation. The cells were counted using the TC10™ Automated Cell Counter (Bio-Rad Laboratories GmbH, Munich, Germany). Afterwards, 1*10^5^–5*10^5^ cells were resuspended in cryopreservation medium containing 70% DMEM F12-Ham, 20% FBS, and 10% DMSO (Sigma-Aldrich) and then stored in liquid nitrogen until pbMEC from all animals had been sampled. For the experimental set-up, pbMEC were thawed and reseeded at 2*10^4^ cells per well of a 6-well plate (Greiner Bio-One GmbH), coated with 2.4 mg/ml Matrigel®, for the immune stimulatory experiments or 1*10^4^ cells per chamber of a 8-well LabTec chamber slide (LAB-Tek, Nunc, GmbH, Langenselbold, Germany) for immunocytochemistry (IC).

### Immune stimulation of pbMEC with formalin inactivated Clostridium Difficile

To calculate the multiplicity of infection (MOI) per cultured cell, three wells per animal served as counting wells. Confluent cells were detached using 0.25% Trypsin-EDTA solution and counted using the TC10™ Automated Cell Counter (Bio-Rad Laboratories GmbH), using life-dead staining with 0.4% trypan blue (Bio-Rad Laboratories GmbH). The mean value of the counted living cells served as the estimated cell count for all other cells used in the experiment. Cell culture replicates of pbMEC were then induced with formalin inactivated *C. diff.* (IDT Biologika GmbH) with a MOI of 70 colony forming units per cultured cell. The MOI was chosen based on the findings of preliminary experiments. A greater MOI was chosen, as compared to reports in the literature, as gram-positive pathogens induce only a weak immune response in pbMEC [12, 16, 17]. To target the immediate, intermediate, and late immune response, pbMEC were treated with *C. diff.* For 6 h, 24 h and 72 h respectively. To obtain representative data, control wells with untreated pbMEC were also sampled in biological triplicates at each time-point (0 h, 6 h, 24 h, and 72 h). To avoid the side effects of antibiotics, antimycotics, and FBS, the cells were supplemented with DMEM/F-12 Ham medium with ITS for 48 h pre-infection. This so-called “infection medium” was refreshed immediately before treatment. After treatment, pbMEC were washed with PBS and further lysed in Qiazol (Qiagen, Hilden, Germany), which was included with the miRNeasy Micro Kit (Qiagen).

### Mycoplasma test

To detect the presence of contaminant mycoplasma species in the cell culture, the PCR Mycoplasma Test Kit (AppliChem GmbH, Darmstadt, Germany) was used according to the manufacturer’s protocol. Cell culture supernatants of each animal were sampled and stored at −80 °C until further processing.

### Immunocytochemistry

pbMEC were cultured on 8-well LabTec chamber Slides (LAB-Tec, Nunc, GmbH, Langenselbold, Germany) to confirm the epithelial character of the cells cultured in 3D cell culture with immunocytochemistry. The IC was conducted as described by Sorg et al. (2013a) and Danowski et al. (2013) [12, 14, 15]. The monoclonal mouse anti-cytokeratin pan antibody clone C-11 (1:400 in PBST, Sigma-Aldrich) was used for cytokeratin staining.

### RNA extraction and reverse transcription

RNA was extracted using the miRNeasy Micro Kit (Qiagen), according to the manufacturer’s protocol with slight modifications. The miRNeasy Micro spin column was incubated for 5 min with buffer RPE after the second addition of buffer RPE to reduce contamination of the RNA with guanidine thiocyanate. The RNA concentration was calculated using a Nanodrop ND-1000 spectrophotometer (Peqlab, Erlangen, Germany). The RNA integrity was analyzed with a 2100 Bioanalyzer on the 6000 nano chips and the RNA 6000 nano Kit (Agilent Technologies, Waldbronn, Germany) according to the manufacturer’s instructions. RNA was stored at −80 °C until further analysis. For reverse transcription of RNA to cDNA, 400 ng of RNA were mixed together with a master mix containing 5× buffer, 0.5 mM dNTPs, 0.5 M oligo-d(T) primers (Fermentas GmbH, St. Leon-Rot, Germany), 2.5 μM random hexamer primers (Invitrogen Life Technologies, Darmstadt, Germany) and 100 U of Moloney murine leukemia virus H(−) reverse transcriptase (Promega, Mannheim, Germany) in a total volume of 20 μl. After reverse transcription, the cDNA was diluted 1:1 to a final volume of 40 μl. RNA isolated from the bovine mammary gland and spleen tissues was used as a positive control. Furthermore, a non-template control (NTC) was included with each 96-well plate (4titude, Wotton, Great Britain) to screen for contamination of the reaction mixture. The RT-PCR reactions were conducted in 96-well plates using a T-Personal Thermocycler (Biometra, Göttingen, Germany) (Annealing: 21 °C, 10 min, transcription phase: 48 °C, 50 min, degrading phase: 90 °C, 2 min). The remaining cDNA was stored at −20 °C.

### RT-qPCR primer design

Bovine specific primer pairs were designed using published bovine nucleic acid sequences retrieved from the National Center for Biotechnology Information gene database (NCBI, National Library of Medicine, Bethesda MD, USA). 68 bovine specific primer pairs were generated (Sigma-Aldrich), among them were 7 primer pairs for the reference genes *GAPDH*, *YWHAZ*, *H3F3A*, *ACTy1*, *18srRNA*, *Cyt8*, *UBB* and respectively 61 primer pairs for target genes coding for proteins involved in inflammatory pathways (Additional file 1: Table S1). For the selection of the panel of genes used in this study, we focused on publications that extensively studied the innate immune response of pbMEC in two-dimensional cell culture in vitro studies [12, 14, 16, 18–21]. Primers were designed using Primer3web version 4.0.0 [22, 23]. The specificity and performance of all primers were tested. All primers had an optimal annealing temperature of 60 °C. Each designed assay was tested using cDNA generated from udder parenchyma tissue, spleen tissue, and pbMEC to confirm tissue-specific gene expression. Furthermore, each qPCR assay was tested for amplification efficiency according to the Minimum Information for Publication of Quantitative Real-Time PCR Experiments (MIQE) guidelines [24]. Only assays with a PCR efficiency >85% were used for subsequent RT-qPCR experiments.

### RT-qPCR measurements

The RT-qPCR analysis was conducted using the BioMark™ HD 96 × 96 system (Fluidigm, San Francisco, CA, USA) as described by Sorg et al. (2013) with slight optimizations [12]. The cDNA was specifically pre-amplified for 16 cycles using 67 primer pairs. The 18srRNA primer pair was excluded from pre-amplification as it was scored as highly expressed gene. In brief, 2 μl of cDNA (10 ng/μl) were pre-amplified in a total volume of 15 μl with a final primer concentration of 25 nM using the iQ Supermix (Bio-Rad) according to the following temperature protocol: activation of polymerase at 95 °C for 3 min, followed by 16 cycles of denaturation at 95 °C for 15 s and 4 min of annealing and extension at 59 °C. The cDNA was diluted 20-fold after the pre-amplification reaction and stored at −20 °C until further analysis. For the determination of the Cq values, 4 BioMark™ 96 × 96 Gene expression (GE) Dynamic Array chips (Fluidigm) were used. The efficiency of all primer assays was tested on the first 96 × 96 GE dynamic array (Fluidigm). Furthermore, each 96 × 96 GE dynamic array contained positive controls, one no transcription control (NTC) and one control sample to test for possible genomic contaminations, called ValidPrime® (TATAA Biocenter, Gothenburg, Sweden). ValidPrime® is a good alternative to avoid the use of reverse transcriptase controls for RT-qPCR analysis, as it tests all samples for the presence of genomic DNA. Two stably expressed samples of the first 96 × 96 GE dynamic array were chosen as between-chip calibrators and, hence, were measured on all four chips. For the sample pre-mix, 2.5 μl SsoFast™ EvaGreen supermix (Bio-Rad), 0.1 μl of ROX (4× diluted, Invitrogen), 0.25 μl of 20× binding dye loading reagent (Fluidigm), 1 μl pre-amplified and 1:20 diluted cDNA and 1.15 μl water were combined to a final volume of 5 μl. The 5 μl assay mix consisted of 2.5 μl of 5 μM primer pairs (final concentration of primers in an individual reaction: 250 nM) and 2.5 μl of 2× GE assay loading reagent (Fluidigm). The sample and assay pre-mix were transferred to the primed 96 × 96 GE dynamic array and then automatically mixed inside the chip with the Fluidigm® IFC controller. The RT-qPCR assay was conducted using the BioMark™ system with the following protocol: 98 °C for 40 s followed by 30 cycles at 95 °C for 10 s and 60 °C for 40 s, followed by melting curve analysis to reveal the specificity of the primer pairs. Fluidigm Real-Time PCR Analysis Software version 4.1.2 (Fluidigm) was used for data handling and analysis. The RT-qPCR reactions were performed according to the MIQE guidelines [24].

### Data pre-processing and data analysis

The qPCR reactions were validated with the Fluidigm Real-Time PCR Analysis Software version 4.1.2 (Fluidigm). Primer pairs with too much missing data were excluded from further analysis (CYP1A1, IL1-B, IL10, CASP1, HP, TAP, LAP, and CCL2). Furthermore, standard curves generated on the first BioMark™ 96 × 96 GE Dynamic Array chip (Fluidigm), were used to determine the efficiency of the primer pairs and the cut-off value for the gene expression data. The dynamic range of the primer assays was tested and the cut-off value was therefore set to 26. The raw data were pre-processed in GenEx Enterprise Version 6 data analysis software (MultiD Analyses AB, Gothenburg, Sweden). Within GenEx values larger than 26 were treated as missing data, the cut-off was set to a Cq-value of 26, and missing data was treated with an offset of “+1”. Furthermore, the genomic background of each sample was evaluated, and an inter-plate calibration was conducted using the mean value of the two inter-plate calibrator samples. The pre-processed Cq values were normalized to the values of a set of seven reference genes, as suggested by the ‘Normfinder’ tool of the GenEx software package (MultiD Analyses AB). Additionally, the normalized Cq values were further normalized to the corresponding reference samples, which were represented by the Cq values of untreated control wells that were sampled at the start of treatment (time point 0 h). The fold changes (2^^(−ΔΔCq)^) were calculated as described by Livak and Schmittgen (2001) [25]. All statistical analyses were conducted using SigmaPlot 12.0 software (Systat Software, Inc., San Jose, CA, USA). Before *p*-values were calculated, the normal distribution of the data sets was confirmed with the Shapiro–Wilk normality test. The signed-rank test was conducted for data not normally distributed. To evaluate the treatment effect of *C. diff.* The ΔCq values of the treated and untreated groups were compared using the paired *t*-test. Significant differences in the gene expression between the different treatment time points (6 h vs. 24 h, 6 h vs. 72 h, 24 h vs. 72 h) were also evaluated using the paired *t*-test. Furthermore, a normal *t*-test was conducted to identify differentially expressed genes between the high and low responder group. Gene expression changes with *p*-values between 0.1 and 0.05 were considered as distinct changes in gene expression, whereas *p*-values below 0.05 were considered as statistically significant changes in gene expression (*p ≤ 0.05, ** *p* ≤ 0.01, *** *p* ≤ 0.001). As no correction for multiple testing was imposed on the p-values, this study has to be considered as explorative study. For the identification of similar gene expression profiles, a cluster analysis with the self-organizing tree algorithm (SOTA) was conducted with the Multi Experiment Viewer software (MeV 4.9.0, TM4) [26].

## Results

### C. Diff. Specific IgA in cow milk

**Fig. 1:**
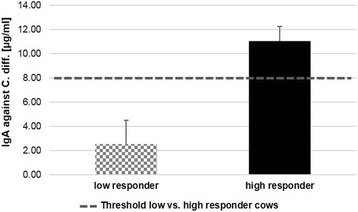
Determination of the specific immunoglobulin A (IgA) content in milk. The animals (*n* = 9) were classified according to their immune response to the *C. diff.* Vaccine into the low (*n* = 4) and high (*n* = 5) responder group. The specific IgA content in milk was measured using a sandwich ELISA, the threshold to distinguish between low and high responder animals was set to 8 μg/ml specific IgA

The IgA content in milk was determined using an IgA ELISA as described above. To distinguish between high and low responder cows, the threshold of *C. diff.* Specific IgA in secreted milk of the immunized animals was set to 8 μg/ml milk. Therefore, four cows were considered as low responder animals with an average specific IgA content of 2.6 μg/ml ± 1.9 μg/ml and five were considered as high responder animals with an average specific IgA content of 11.1 μg/ml ± 1.2 μg/ml milk (*p* ≤ 0.001) (Fig. 1).

### pbMEC cell culture – IC and mycoplasma test

**Fig. 2:**
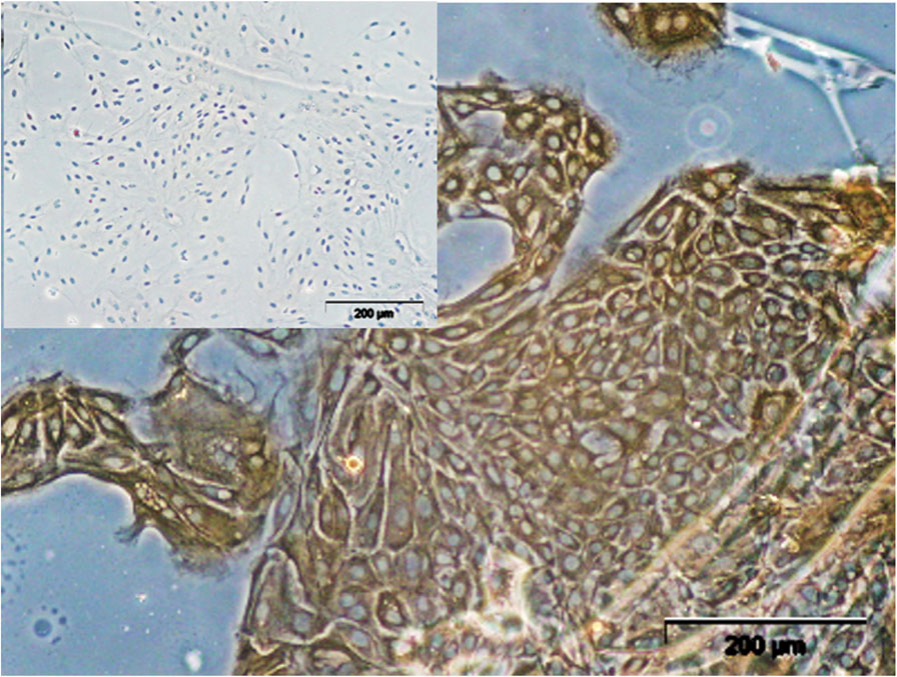
Light microscopy of pbMEC isolated from fresh milk immunostained against cytokeratin (the inset shows the negative control, magnification ×200). pbMEC were cytokeratin-positive and showed the characteristic cobblestone-like morphology of the cells

IC analysis was conducted to confirm the epithelial character of the 3D cultured pbMEC. All cultured cells were cytokeratin-positive and showed a typical cobblestone-like morphology, which is characteristic for pbMEC (Fig. 2). Therefore, cross-contamination with other cells was excluded. Furthermore, all cells were mycoplasma-free (PCR Mycoplasma Test Kit, AppliChem GmbH, Darmstadt, Germany).

### Quality control of the extracted RNA and the RT-qPCR assays

The quality of the extracted RNA was assessed as described before. In brief, the total RNA yield was determined using a Nanodrop ND-1000 spectrophotometer. The overall RNA yield was 335.21 ng/μl ± 15.00 ng/μl (*n* = 314). The RNA integrity which was analyzed with the 2100 Bioanalyzer on the 6000 Nano chips was measured for 70 RNA samples that were randomly collected over all 4 time-points. An average RIN value of 9.94 ± 0.13 (*n* = 70) could be determined, indicating very good integrity of all RNA samples, as the highest possible RIN is 10. Furthermore, the designed qPCR assays were tested for their efficiency in qPCR reactions according to the MIQE guidelines [24], using standard curves of serial diluted sample material. The performance of the assays was tested on the BioMark™ 96 × 96 GE dynamic array. Assays with poor PCR amplification efficiency were excluded from further analysis (CYP1A1, IL1-B, IL10, CASP1, HP, TAP, LAP, and CCL2). Analyses of the remaining 60 qPCR assays resulted in an average *r* ^2^-value of 0.97 ± 0.01 (*n* = 60) and an average PCR efficiency of 1.14 ± 0.025 (*n* = 60), indicating that the PCR efficiency of the primer pairs was between 90% - 114%.

### Effect of the C. Diff. Treatment on the gene expression within the high responder cows

According to the Ig yield obtained in the milk, the vaccinated cows showed a rather diverse immune response. Therefore, a gene expression profiling method was applied to identify molecular biomarkers of innate immunity of cows with a fast and efficient immune response. A detailed listing of the fold changes in gene expression and the calculated *p*-values that were determined by the paired *t*-test for the treatment effect and the time effect can be found in “Additional file 2: Table S2”. For the identification of differences within the expression profiles of low and high responder animals, a cluster analysis with the self-organizing tree algorithm (SOTA) was conducted applying the Multi Experiment Viewer software (MeV 4.9.0, TM4) [26]. The analysis was done based on mean centered fold change values. Genes with higher fold changes than the mean are highlighted in green, whereas fold changes below the mean fold change are highlighted in red. With this method, the time course of gene expression changes within the high and low responder groups could be illustrated whereby the genes were clustered concerning their early, intermediate or late gene expression.

### SOTA analysis of RT-qPCR data

**Fig. 3:**
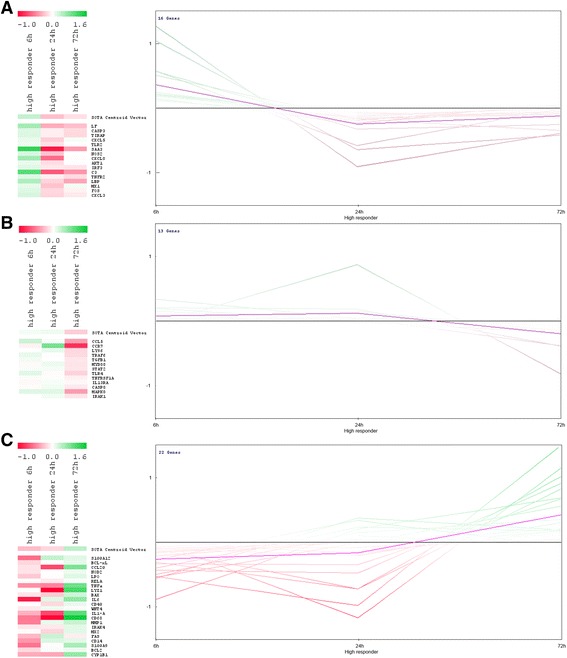
Self-organizing tree algorithm (SOTA) analysis revealed early (16 genes) (**a**), intermediate (13 genes) (**b**)**,** and late (22 genes) (**c**) expression profiles of genes stimulated with *C. diff*. in the high responder group. Sample fold changes compared to the mean fold change of the whole group are highlighted in the SOTA dendrogram (left) as well as in the SOTA expression profile (right). Fold changes greater than the mean fold change are highlighted in green, whereas fold changes below the mean fold change are highlighted in red

Genes coding for FcRn and pIGR were excluded from the SOTA analysis of the high and low responder groups because of no contribution to the scientific question. SOTA analysis of the high responder group revealed three clusters, one composed of early induced genes (Fig. 3a), a second composed of the intermediate induced genes (Fig. 3b), and a third composed of genes that were mostly induced at 72 h after immune stimulation (Fig. 3c). The first cluster contained 16 genes, which were induced early after immune stimulation, which included some really strongly induced genes coding for the acute phase protein SAA3, the antimicrobial peptide lactoferrin (LF), the complement component C3, the components of the Toll-like receptor (TLR) pathway LBP, TLR2, and TIRAP, and the chemokines CXCL5, CXCL3, and CXCL8 (Fig. 3a). The effect of *C. diff. Treatment* was statistically evaluated using the paired *t*-test (Additional file 2: Table S2). The gene expression levels of CXCL8, CXCL3, and TIRAP were up-regulated in response to immune stimulation. The time-dependent effects of immune stimulation on gene expression are shown in the SOTA dendrogram (Fig. 3a) as well Additional file 2: Table S2.

Within the second cluster, 13 genes were detected, which were rather early (6 h) and intermediately (24 h) induced (Fig. 3b). Genes coding for the chemokines CCL5, CCR7, and IL13RA, the components of the TLR pathway (i.e., TLR4, LY96, MYD88, TRAF6, and IRAK1), and the gene coding for MAPK8 were strongly induced either early or intermediately. According to the paired *t*-test results, the gene expression of genes coding for IRAK1 and TRAF6 were differentially up-regulated in response to immune stimulation (Fig. 3b; Additional file 1: Table S1). Regulation of gene expression at different treatment time points in this cluster was directly observed with the SOTA dendrogram and the expression graph presented in Fig. 3. These results were verified with a paired *t*-test (Additional file 2: Table S2).

Within the third cluster, 22 intermediate to late induced genes were detected, which included strongly induced genes coding for TNFα, CD68, CD14, and CYP1B1. Furthermore, the genes coding for the so-called “danger-associated molecular pattern molecules” S100A9 and S100A12, the antimicrobial peptides lysozyme 1 (LYZ1) and lacto-peroxidase (LPO), the chemokines and inflammatory cytokines CCL20, IL6, and IL1-A, the components of the apoptotic pathway (e.g., FAS), the scavenger receptor CD68, and the gene coding for MMP1 were differentially induced in response to *C. diff.* Stimulation (Additional file 2: Table S2). This trend was also observed in the SOTA dendrogram and expression profile (Fig. 3c), where again the temporal regulation of the gene expression of immune-related genes was clearly determined (Fig. 3c; Additional file 2: Table S2).

### Comparison of the gene expression pattern of low and high responder cows during different treatment time-points

**Fig. 4:**
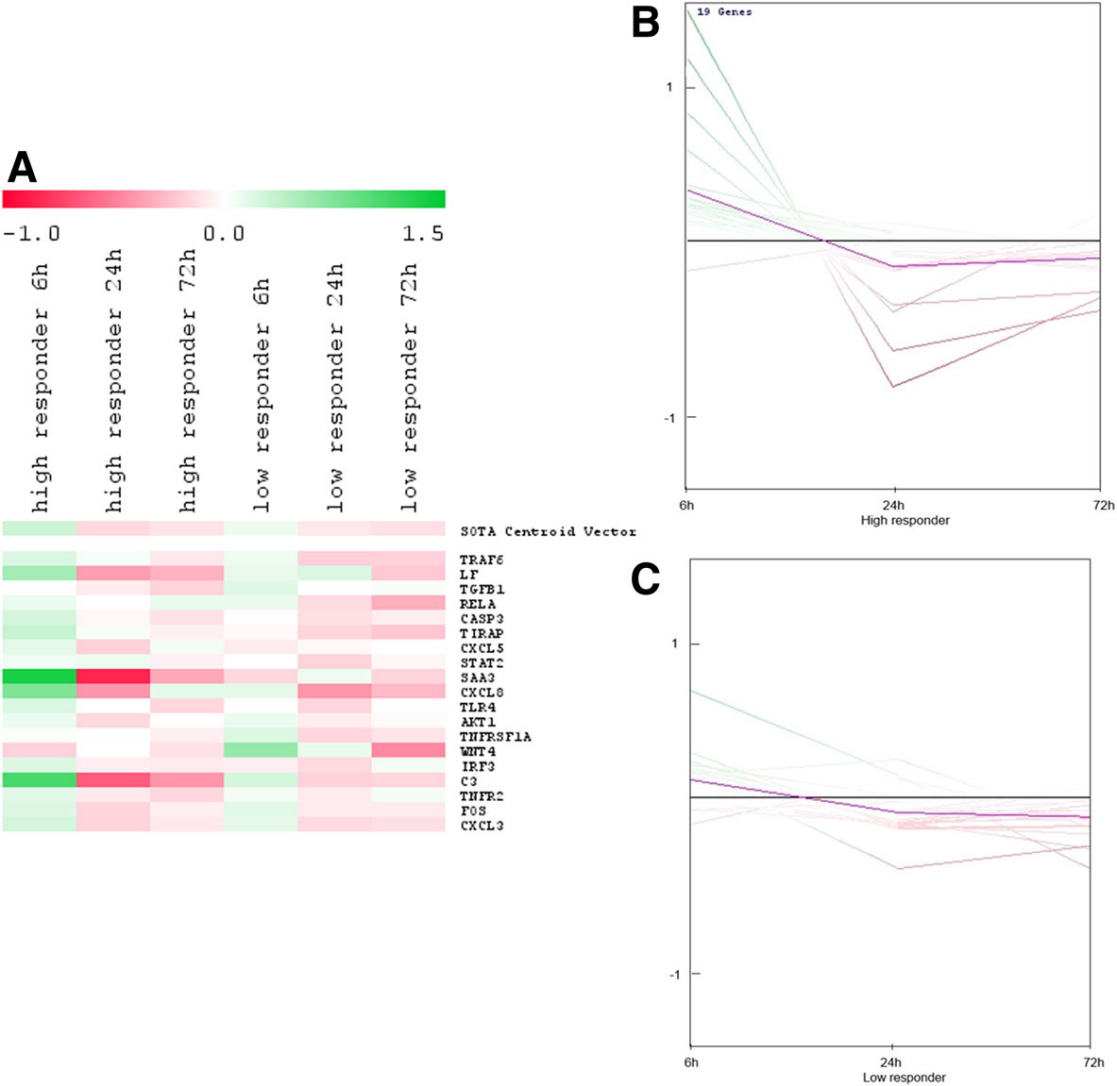
Differences in the gene expression of immediate early expressed genes (19 genes) between the high (**b**) and low (**c**) responder group. The self-organizing tree algorithm dendrogram (**a**), as well as the expression profile for the high responder (**b**) and low responder (**c**) animals indicated differentially gene expression within both groups

A direct comparison of the gene expression pattern of high and low responder cows indicated a distinct greater induction of the gene expression within the high responder group during all three-time points. Genes were clustered together according to the induction time of the immune response. The first cluster consisted of 19 genes that were early expressed. The SOTA dendrogram (Fig. 4a) was used to identify genes in the high and low responder groups that were induced in response to *C. diff.* Stimulation, which showed that expression levels of some genes were lower in the low responder group (Figs. 4a, b, and c). The potential lower induction of *gene expression* in the *low responder* group is clearly indicated by the SOTA expression graphs (Figs. 4b and c). Most of the early induced genes within the *high responder* group showed a more distinct up-regulation after 6 h of immune stimulation and a strong down-regulation after 24 h. In the *low responder* group, however, only a few of the 19 genes showed a greater fold change, as compared to the mean fold change of the whole group, *and* the *gene expression within the low responder* group *declined* after 24 h. The strongly induced genes in the *high responder* group included those coding for the antimicrobial peptide LF, the chemokines CXCL8, CXCL5, and CXCL3, the acute phase protein SAA3, and the complement component C3. Furthermore, genes coding for components of the TLR pathway (i.e., TIRAP, TRAF6, and RELA) were differentially induced in the *high responder* group, as compared to the *low responder* group, according to the results of a normal *t*-test (Table 1; Fig. 4 and Additional file 2: Table S2).

**Table 1:** Differences in gene expression of high (*n* = 5) and low (*n* = 4) responder cows, as determined by a normal *t*-test

Time point			
Genes	*C. diff.* 6 h^a^	*C. diff.* 24 h^a^	*C. diff.* 72 h^a^
	Low vs. High^b^	Low vs. High^b^	Low vs. High^b^
TLR pathway			
*LY96*		***	**
*CD14*	+	***	*
*MYD88*		+	
*TIRAP*	*	**	***
*TRAF6*		+	
*IRAK4*		*	
*IRAK1*		+	
*RELA*		**	
Chemokines			
*CCL5*	+	*	
*CXCL5*	+		
*CXCL8*	+		+
*IL13RA*		*	+
Inflammatory cytokines			
*IL1-A*			+
*IL6*	**	+	*
Acute phase proteins/danger associated molecular pattern molecules			
*S100A9*			*
*S100A12*			*
Antimicrobial peptides			
*LYZ1*			**
*LPO*			*
Apoptosis			
*FAS*	*	**	*
*TNFRSF1A*		+	
*CASP8*		**	+
*CASP3*	*	*	
*BAX*		**	
*BCL-2*	+		*
Scavenger Receptor			
*CD68*	**	*	
*CD40*	**	**	*
JAK-STAT signaling			
*STAT2*		**	
MAPK signaling			
*MAPK 8*	**	*	
Others			
*MMP1*		*	**
*MX2*		+	
*NOD2*			*
*AKT1*		*	
*WNT4*			+

^a^Treatment time with *C. diff.* in hours

^b^low responder animals versus high responder animals

*p ≤ 0.05, ** *p* ≤ 0.01, *** *p* ≤ 0.001

+distinct changes (0.01 ≤ *p* < 0.05)

**Fig. 5:**
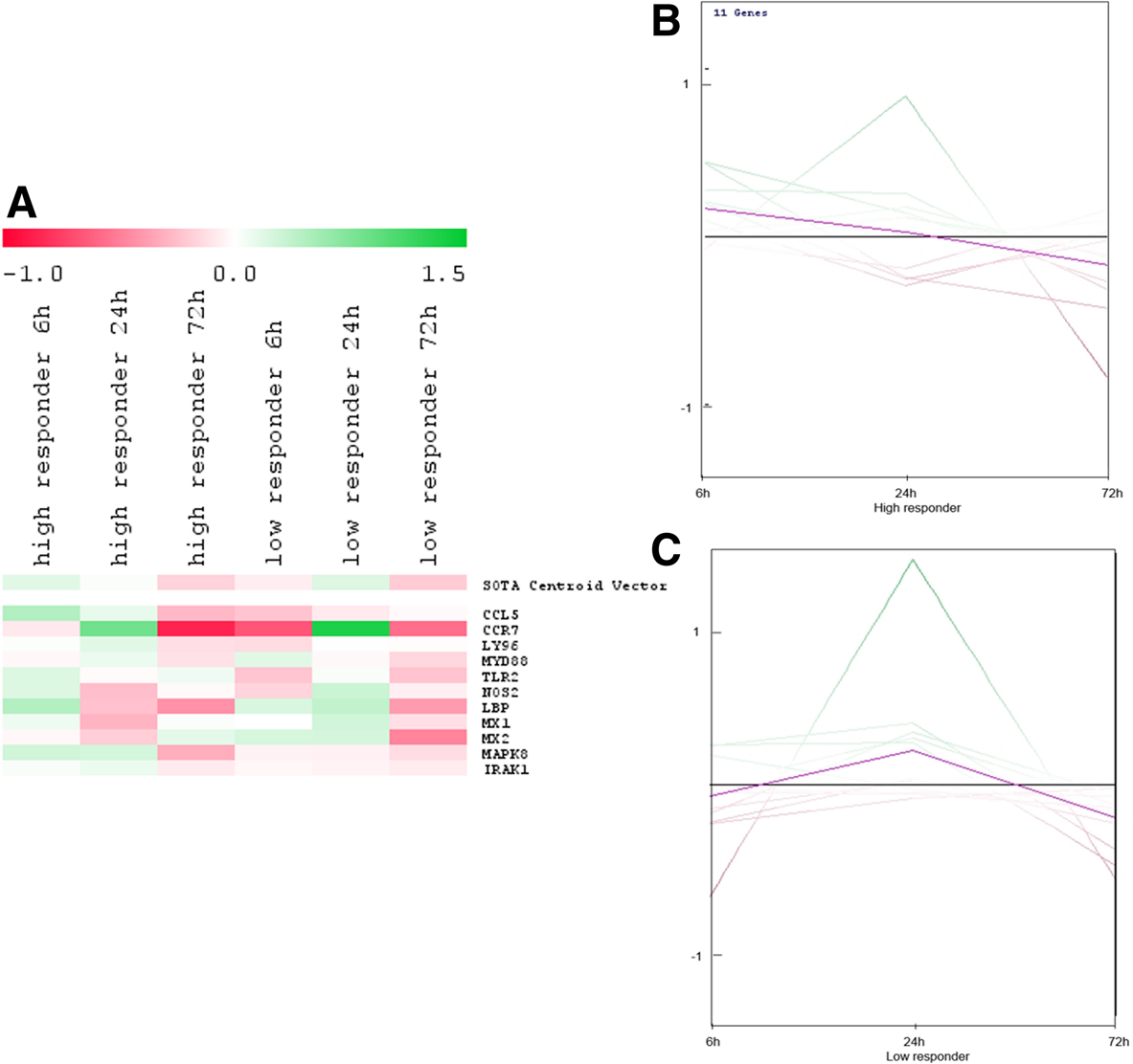
Differences in the gene expression of immediate early and intermediate early expressed genes (11 genes) in the high (**b**) and low (**c**) responder group. Throughout the SOTA dendrogram (**a**), as well as the expression profiles for the high (**b**) and low (**c**) responder group, indicated differences in gene expression pattern within the high and low responder group

Eleven genes in the second cluster were induced either early or intermediately. The dendrogram and expression profile (Figs. 5a and b) clearly indicated that genes within this cluster tended to be up-regulated in the high responder group after 6 h, with the exception of the gene coding for the chemokine receptor CCR7 (Figs. 5a and b). This accounts for an early as well as prolonged induction of the expression of genes coding for important chemokines, like CCL5, and components of the TLR pathway, like LY96, MYD88, TLR2, and IRAK1. Most of these genes, however, were down-regulated 6 h post-immune stimulation in the low responder group and were only induced after 24 h. Some genes, such as those coding for NOS2, LBP, MX1, and MX2 were down-regulated in the high responder group after 24 h, but were induced after this period of time within the low responder group, indicating differences in gene regulation between the groups. By contrast, the genes coding for CCL5, IRAK1, and MAPK8 were up-regulated in the high responder group, but down-regulated in the low responder group. The results of a normal *t*-test (Table 1) revealed distinct and differential up-regulation of the gene expression for LY96, MYD88, IRAK1, CCL5, and MAPK8 between the high and low responder groups (Table 1; Fig. 5; Additional file 2: Table S2).

**Fig. 6:**
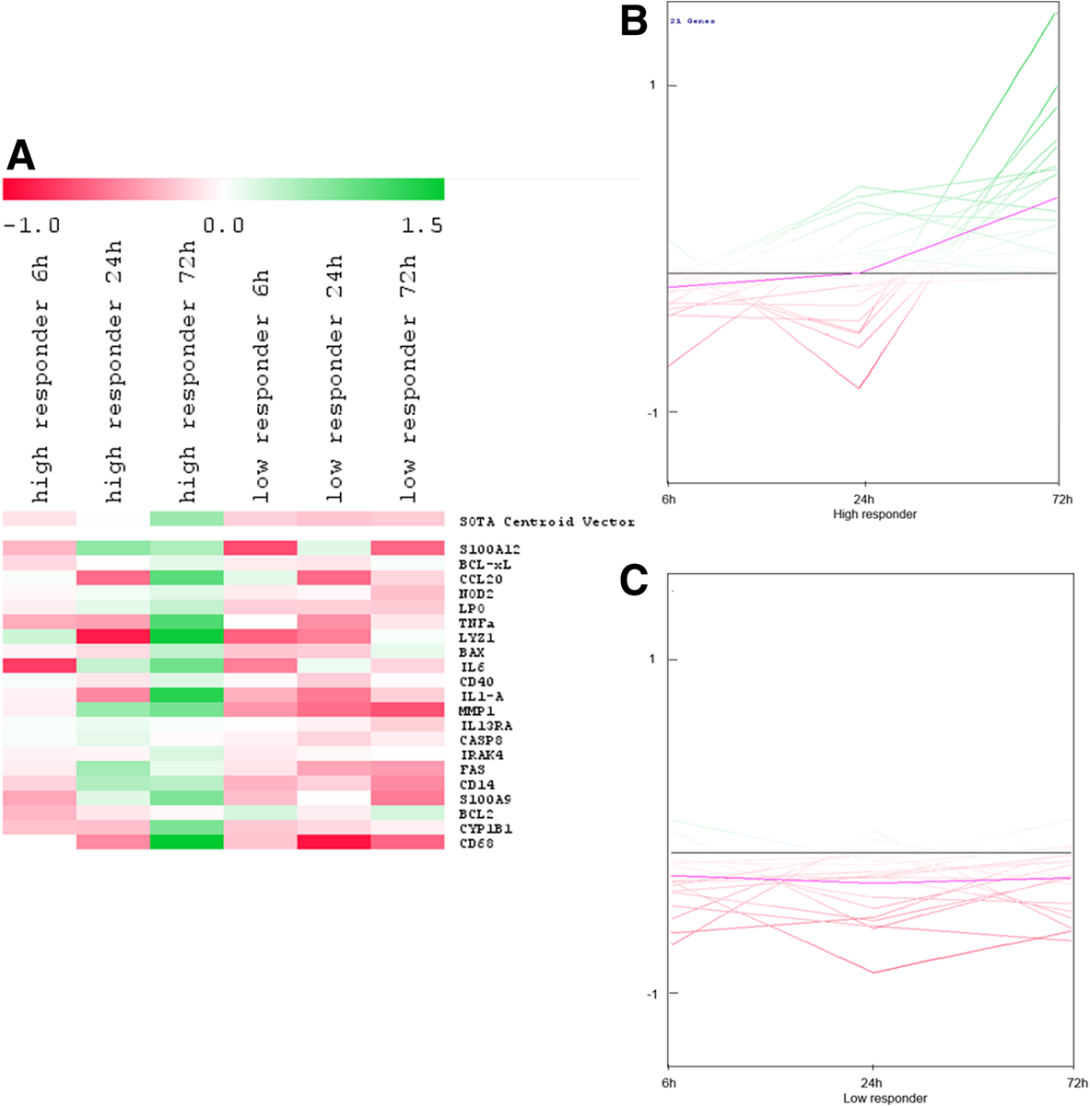
Differences in the gene expression of late induced genes (21 genes) in the high (**b**) and low (C) responder group. The SOTA dendrogram (**a**) as well as the expression profiles for the high (**b**) and low (**c**) responder group, indicated differences in gene expression pattern within the high and low responder group

The third cluster consisted of 21 genes that were induced after 72 h and partly after 24 h of immune stimulation. The SOTA dendrogram revealed that those genes were especially induced in the high responder group after 72 h, as observed by a comparison of color coding with the low responder group at the same time points (Fig. 6a). However, those genes were not induced earlier in the low responder group. The pbMEC in the low responder group hardly showed any induction of immune-related genes after *C. diff.* Stimulation (Table 1). Differentially up-regulated late expressed genes included some that coded for chemokines and inflammatory cytokines, like IL6, IL1-A, and IL13RA, as well as those coding for the danger-associated molecular pattern molecules S100A9 and S100A12, the antimicrobial peptides LYZ1 and LPO, the components of the TLR pathway, like CD14, the pro-apoptotic factors FAS, CASP8, and BAX, and those coding for CD68, CD40, MMP1, and NOD2. Changes in gene expression profiles over-time are depicted in the expression graphs (Table 1; Fig. 6).

## Discussion

The gram-positive pathogen *C. diff.* Was chosen in this study as it causes severe CDAD in those with suppressed immunity and the elderly [3]. Treatment of CDAD with immune milk can offer some significant advantages, such as maintenance of the healthy commensal gut microbiota and the prevention of the formation of resistant bacteria due to the use of natural polyclonal animal-derived antibodies. The pathogen-specific polyclonal IgA can specifically neutralize *C. diff.* and minimize the relapse rate and the number of antibiotic treatments.

After stimulation with *C. diff.*, the gene expression profile of pbMECs extracted from milk was compared between high and low responder cows. The milk of high responder cows (*n* = 5) had high amounts of specific IgA and the animals showed a fast immune response, whereas milk of low responder cows (*n* = 4) had lower antibody concentrations after repeated immunization. The term “fast immune response” hereby refers to a rapid increase in the amount of specific IgA in milk after immunization. The aim of this study was to establish a defined gene expression pattern or a special set of genes of chemokines, immune receptors, and acute phase proteins to serve as molecular biomarkers for the pre-selection of cows before immunization to maximize immune milk production. pbMEC were chosen to screen for gene expression responses to antigen exposure, as it is known that bovine mammary epithelial cells play important roles in the bovine mammary gland [11]. The rather low changes in the gene expression levels were expected, as Strandberg et al. (2005), Griesbeck-Zilch et al. (2008), and Sorg et al. (2013) have already reported that gram-positive pathogens provoke only a weak innate immune response [12, 21, 27,].

### TLR pathway

Strandberg et al. (2005) reported that the innate host defense of pbMEC is dependent on germline-encoded receptors that recognize conserved structures expressed by a wide variety of microbes [27]. Since pbMEC express TLRs on the cell surface [16], these cells should be able to recognize the gram-positive pathogen *C. diff.* Upon recognition of the bacterial cell wall component lipoteichoic acid through the pattern recognition receptors CD14, TLR2, and TLR4 [28]. The results of the present study showed that gene expression of TLR2 and TLR4 in response to bacterial stimulation was mainly unaffected in the low and high responder groups. These findings are in accordance with the results reported by Strandberg et al. (2005) [27], who postulated that pbMEC contain a fully functional and constitutively active TLR signaling pathway, which is immediately responsive to a bacterial challenge, so that the gene expression of the receptors was not responsible for the inefficient activation of NFkB and, hence, transcription of cytokines, but rather deficits in the downstream signaling pathways [27]. However, the high responder group showed a distinct and statistically significant greater gene expression of CD14, as compared to the low responder group, which is in accordance with the findings of Lutzow et al. (2008) [29]. To identify differences in the activation and downstream signaling cascades in response to *C. diff.*-stimulation between the high and low responder groups, the gene expression levels of LY96, LBP, CD14, MYD88, TIRAP, TRAF6, IRAK4, IRAK1, and RELA were assessed. The results showed significant and distinct changes in the expression levels of genes coding for LY96, CD14, TIRAP, IRAK1, and RELA, especially in the high responder group. Furthermore, the expression levels of MYD88, TRAF6, LY96, CD14, TIRAP, and RELA were significantly greater in the high responder group, as compared to the low responder group, which was in agreement with the findings of Strandberg et al. (2005) [27] that deficits in the downstream signaling pathways were responsible for the relatively low expression of RELA, which is also known as the NF-kappa-B p65 subunit.

### Chemokine activation

Upon activation, NF-kB translocates into the nucleus and initiates transcription of a variety of pro-inflammatory factors, such as chemokines and cytokines, as well as genes associated with cell survival and proliferation [30]. Targets also include adhesion molecules, acute phase proteins like SAA-proteins, and inducible enzymes [30, 31]. This effect was observed in the gene expression of prominent chemokines between the high and low responder groups. The gene expression of CXCL8, CCL5, CXCL5, IL6, IL1-A, and IL13RA were distinctly greater in the high responder group. Especially, CXCL8, which is a major initiator of the inflammatory response, has been shown to be essential for the immediate recruitment of leukocytes into the bovine mammary gland and, hence, is responsible for the elimination of invading pathogens [27, 32]. CXCL8 gene expression was substantially greater in the high responder fibroblasts after stimulation of the cell culture with LPS. Additionally, a study by Griesbeck-Zilch et al. (2008) also showed a significant and early induction of CCL5 gene expression after stimulation of pbMEC with *S. aureus* [21]. Furthermore, Lahouassa et al. (2007) showed that bMEC are able to produce and release chemokines, even without up-regulation of the anti-inflammatory cytokine IL10, which we were unable to measure in the present study [33]. As the genes coding for chemokines and inflammatory cytokines were particularly more strongly induced within the high responder group, it could be possible that initiation of the inflammatory reaction and the recruitment of other immune cells to the site of infection was more efficient in the high responder group.

### Gene expression pattern of antimicrobial peptides

Activation of the transcription factor NFĸB is also known to induce gene expression and production of the antimicrobial peptides LYZ1, LPO, and LF. Normally, antimicrobial peptides are constitutively expressed, even if no direct bacterial stimuli is present. These peptides are mostly constitutively expressed in cells, such as epithelial cells, which are consistently exposed to bacteria. For example, LF shows a bacteriostatic effect through its capability to bind iron, which is essential for bacterial growth [15]. However, in contrast to the report by Griesbeck-Zilch et al. (2008), no up-regulation in LF gene expression was detected in either of the treatment groups [21]. Lysozyme is also a bactericidal protein that cleaves peptidoglycans of the cell wall of gram-positive and gram-negative bacteria. The third antimicrobial peptide analyzed in this study was LPO, which is able to kill or inhibit bacteria in the presence of thiocyanate and hydrogen peroxide [34]. In the present study, significant induction of the gene expression of LPO and LYZ1 was observed only in the high responder group. Furthermore, the gene expression levels of LPO and LYZ1 were significantly greater in the high responder group, as compared to the low responder group.

### Danger associated molecular pattern molecules

The acute phase proteins S100A12 and S100A9 also participate in the regulation of inflammatory processes, as well as the induction of cytokine and chemokine production. The significantly greater gene expresison levels in the high responder group compared to the low responder group could, therefore, together with the chemokines, also contribute to greater activation of immune cells, resulting in a stronger and faster adaptive immune response than in the low responder group [35, 36]. The induction of S100A12 gene expression through gram-positive pathogens has already been reported by Lutzow et al. (2008), Sorg et al. (2013), and Günther et al. (2009), which prompted the question as to whether these molecules are involved in the initial response to bacterial infection [11, 12, 29].

### Apoptosis related genes

Apoptosis is an important biochemical process responsible for the proper development and function of the immune system. It has already been shown that apoptosis of bovine mammary epithelial cell lines and primary bovine epithelial cells occurs in response to *S. aureus* infection [37]. Considering the induction of apoptosis, the pbMEC of the high responder group also showed significantly stronger induction of the pro-apoptotic genes Bax, FAS, CASPASE 8, and CASPASE 3 post-infection, as compared to the low responder group. This finding could indicate that the cells in the high responder group were subjected to stronger apoptotic events.

## Conclusions

When the expression patterns of genes involved in the TLR signaling pathway and those coding for effector molecules were compared between the low and high responder group, it seems that induction of the innate immune response was quicker in the high responder animals. The greater expression levels of genes involved in the TLR pathway, cytokines, and antimicrobial peptides in pbMEC of the high responder group could be advantageous for the recruitment and activation of immune cells, resulting in a stronger and faster adaptive immune response than in the low responder group, which in turn leads to a faster induction of antibody producing B-cells and to greater Ig concentrations in milk. It might be possible that the gene expression pattern of the pbMEC during infection together with the gene expression pattern of the bovine lymphocytes is the key to the discovery of new molecular biomarkers to identify cows with an effective immune response and greater amounts of Igs produced in milk. The data obtained from cell culture studies with pbMEC, will be correlated with the gene expression pattern of bovine lymphocytes in our next publication. So, far, genes coding for components of the TLR pathway (LY96, CD14, TIRAP, and RELA), the chemokines CXCL8, CCL5, and CXCL5, the inflammatory cytokines IL6 and IL1-A, the antimicrobial peptides LYZ1 and LPO, and the danger-associated molecular pattern molecules S100A9 and S100A12 appear to be promising as potential candidates for molecular markers, as all were differentially expressed between the low and high immunoglobulin responder group.

## Additional files

Additional file 1: **Table S1.** Primer for RT-qPCR measurements. All primer names, sequences and the NCBI reference sequence number are presented in Additional file 1. (DOCX 45 kb)

Additional file 2: **Table S2.** Fold changes in gene expression upon C. diff. Treatment - statistical evaluation of the treatment and time-effect with a paired t-test. High responder (*n* = 5), low responder (*n* = 4). (DOCX 40 kb)

## Abbreviations

18SrRNA: 18S ribosomal RNA gene
ACTG1: Actin, gamma 1
AKT1: V-akt murine thymoma viral oncogene homolog 1
BAX: BCL2-associated X protein
BCL-2: B-cell CLL/lymphoma 2
*C. diff.*: *Clostridium difficile*
C3: Complement component 3
CASP1: Caspase 1
CASP3: Caspase 3
CASP8: Caspase 8
CCL2: Chemokine (C-C motif) ligand 2
CCL20: Chemokine (C-C motif) ligand 20
CCL5: Chemokine (C-C motif) ligand 5
CCR7: Chemokine (C-C motif) receptor 7
CD14: CD14 surface receptor
CD40: CD40 surface receptor
CD68: CD68 surface receptor
cDNA: Complementary DNA
Cq: Cycle of quantification
ctr: Control
CXCL3: Chemokine (C-x-C motif) ligand 3
CXCL5: Chemokine (C-x-C motif) ligand 5
CXCL8: Chemokine (C-X-C motif) ligand 8
CYP1A1: Cytochrome P450, family 1, subfamily A, polypeptide 1
CYP1B1: Cytochrome P450, family 1, subfamily B, polypeptide 1
DMEM/F-12 HAM: Dulbecco’s modified eagle’s medium nutrient mixture f-12 ham
DMSO: Dimethyl sulfoxide
DNA: Deoxyribonucleic acid
dNTP: Deoxynucleoside triphosphate
*E. coli*: *Escherichia coli*
EDTA: Ethylenediaminetetraacetic acid
FAS: Fas cell surface death receptor
FBS: Fetal bovine Serum
FcRN: IgG Fc receptor
GAPDH: Glyceraldehyd-3-phosphate dehydrogenase
H_2_SO_4_: Sulfuric acid
H3F3A: H3 histone, family 3A
HBSS: Hank’s balanced salt solution
HC: Hydrocortisone
HP: Haptoglobin
HRP: Horse radish peroxidase
IgA: Immunoglobulin A
IL10: Interleukin 10
IL13RA: Interleukin 13 receptor, alpha 1
IL1-A: Interleukin 1, alpha
IL1-B: Interleukin 1, beta
IL6: Interleukin 6
IRAK1: Interleukin-1 receptor-associated kinase 1
IRAK4: Interleukin-1 receptor-associated kinase 4
IRF3: Interferon regulatory factor 3
ITS: Insulin/Transferrin/Sodium selenite solution
KRT8: Cytokeratin 8
LAP: Lingual antimicrobial peptide
LBP: Lipopolysaccharide binding protein
LF: Lactoferrin
LPO: Lactoperoxidase
LPS: Lipopolysaccharide
LTA: Lipoteichoic acid
LY96: Lymphocyte antigen 96
LYS: L-Lysine
LYZ1: Lysozyme 1 K
MAPK1: Mitogen-activated protein kinase 1
MAPK8: Mitogen-activated protein kinase 8
MIQE: Minimum information for publication of quantitative real-time PCR experiments
M-MLV: Moloney murine leukemia virus
MMP1: Matrix metallopeptidase 1
MOI: Multiplicity of infection
mRNA: Messenger RNA
MX1: Myxovirus (influenza virus) resistance 1, interferon-inducible protein p78 (mouse)
MX2: Myxovirus (influenza virus) resistance 2 (mouse)
MYD88: Myeloid differentiation primary response gene 88
NaHCO_3_: Natrium hydrogen carbonate
NCBI: National Library of Medicine
NFκB: Nuclear factor κb
NOS2: Nitric oxide synthase 2, inducible
NTC: Non-template control
PAMP: Pathogen associated molecular pattern
pbMEC: primary bovine mammary epithelial cells
PBS: Phosphate Buffered Saline
PBS-T: PBS-Tween20
PenStrep: Penicillin/Streptomycin
pIGR: Polymeric immunoglobulin receptor
qPCR: Quantitative polymerase chain reaction
RELA: V-rel reticuloendotheliosis viral oncogene homolog A (avian) (NF-kappa-B p65 subunit)
RIN: RNA integrity number
RNA: Ribonucleic acid
rpm: Revolutions per minute
rRNA: Ribosomal RNA
RT: Reverse transcription/room temperature
RT-qPCR: Reverse transcription quantitative polymerase chain reaction
S100A12: S100 calcium binding protein A12
S100A9: S100 calcium binding protein A9
*S. aureus*: *Staphylococcus aureus*
SAA3: Serum amyloid A3
SCC: Somatic cell count
SEM: Standard error of the mean
SOTA: Self-organizing tree algorithm
STAT2: Signal transducer and activator of transcription 2
TAP: Tracheal antimicrobial peptide
TGFβ1: Transforming growth factor, beta 1
TIRAP: TCDD-inducible poly(ADP-ribose) polymerase
TLR: Toll-like receptor
TLR2: Toll-like receptor 2
TLR4: Toll-like receptor 4
TNF: Tumor necrosis factor
TNFR2: Tumor necrosis factor receptor 2
TNFRSF1A: Tumor necrosis factor receptor superfamily, member 1A
TNFα: Tumor necrosis factor α
TRAF6: TNF receptor-associated factor 6, E3 ubiquitin protein ligase
UBB: Ubiquitine B
WNT4: Wingless-type MMTV integration site family member 4
YWHAZ: Tyrosine 3-monooxygenase / tryptophan 5-mono-oxygenase activation protein zeta polypeptide
YY1: Yin Yang 1
