# Supplementary material for: Gene expression profiling in pbMEC – in search of molecular biomarkers to predict immunoglobulin production in bovine milk
Source: BMC Vet Res. 2017 Nov 29;13:369. doi: 10.1186/s12917-017-1293-z (PMC5707921; doi:10.1186/s12917-017-1293-z)
Supplement: Supplementary file 1 — Table S1. Primer for RT-qPCR measurements. All primer names, sequences and the NCBI reference sequence number are presented in Additional file 1. (DOCX 45 kb) [file 12917_2017_1293_MOESM1_ESM.docx]

*Additional File 1*

Gene expression profiling in pbMEC – in search of molecular biomarkers to predict immunoglobulin production in bovine milk

M. Hillreiner 1_,_ C. Schmautz 1, I. Ballweg ^1^, V. Korenkova ^2^, MW. Pfaffl ^1^ and H. Kliem 1

1 Chair of Animal Physiology and Immunology, Technische Universität München, Weihenstephaner Berg 3, 85354 Freising, Germany

2 Quantitative and Digital PCR Core Facility, Institute of Biotechnology CAS, v. v. i. BIOCEV Center, 252 50 Vestec by Prague, Czech Republic

Corresponding author: Heike Kliem. Email: kliem@wzw.tum.de

*Table S1:* Primer for RT-qPCR measurements

| Gene name | NCBI reference sequence number | Primer sequence (5’ -> 3’)  Forward  Reverse | L^1^ [bp] |
| --- | --- | --- | --- |
| ***TLR pathway*** |  |  |  |
| Toll-like receptor 2 (*TLR2*) | NM_174197.2 | CATTCCTGGCAAGTGGATTATC  GGAATGGCCTTCTTGTCAATGG | 201 |
| Toll-like receptor 4 (*TLR4*) | NM_174198.6 | TGCTGGCTGCAAAAAGTATG  TTACGGCTTTTGTGGAAACC | 213 |
| Lymphocyte antigen 96 (*LY96*) | NM_001046517.1 | TGTTTCAATACGTTCTGAGCCC  TCAGTGTTCCCCTCGATGG | 300 |
| Lipopolysaccharide binding protein (*LBP*) | NM_001038674.2 | TCCCAGTTGCTTTCCTTGCT  GCGGAAGGACTTGGTGTTCT | 194 |
| CD14 molecule (*CD14*) | NM_174008.1 | GCAGCCTGGAACAGTTTCTC  ACCAGAAGCTGAGCAGGAAC | 124 |
| Myeloid differentiation primary response gene (*MYD88*) | NM_001014382.2 | CTGCAAAGCAAGGAATGTGA  AGGATGCTGGGGAACTCTTT | 122 |
| TCDD-inducible poly(ADP-ribose) polymerase (*TIRAP*) | NM_001206048.1 | TAGTGCAGCCTCCTTCTCCT  AACCCCATCAAGTGAGCCAG | 176 |
| TNF receptor-associated factor 6, E3 ubiquitin protein ligase (*TRAF6*) | NM_001034661.2 | GGACTGCAGCAAAAGACGAC  CTTCCCGCAAAGCCATCAAG | 156 |
| Interleukin-1 receptor-associated kinase 4 (*IRAK4*) | NM_001075998.1 | ACAGCATCAACATACGTGCG  GGTGCCCCAGTCAAACAGTA | 213 |
| Interleukin-1 receptor-associated kinase 1 (*IRAK1*) | NM_001040555.1 | GCCGCCCAGATCTACAAGAA  TAGGAGTTCTCTTGCGGGGA | 233 |
| V-rel reticuloendotheliosis viral oncogene homolog A (avian) (NF-kappa-B p65 subunit)  (*RELA*) | NM_001080242.2 | ACAGCTTTCAGAACCTGGGG  GACGGCATTCAGGTCGTAG | 140 |
| ***Complement system*** |  |  |  |
| Complement component 3 (*C3*) | NM_001040469 | AAGTTCATCACCCACATCAAG  CACTGTTTCTGGTTCTCCTC | 191 |
| ***Chemokines*** |  |  |  |
| Chemokine (C-C motif) ligand 2 (*CCL2*) | NM_174006.2 | TCTCGCTGCAACATGAAGGT  TATAGCAGCAGGCGACTTGG | 121 |
| Chemokine (C-C motif) ligand 5 (*CCL5*) | NM_175827.2 | TCCATGGCAGCAGTTGTCTT  TTCAGGTTCAAGGCGTCCTC | 129 |
| Chemokine (C-C motif) ligand 20 (*CCL20*) | NM_174263.2 | CTTGTGGGCTTCACACAGC  GTTTCACCCACTTCTTCTTTGG | 115 |
| Chemokine (C-x-C motif) ligand 5 (*CXCL5*) | NM_174300.2 | TTGTGAGAGAGCTGCGTTGT  CCAGACAGACTTCCCTTCCA | 150 |
| Interleukin 8 (*CXCL8*) | NM_173925.2 | AAGAATGAGTACAGAACTTCGATGC  GTTTAGGCAGACCTCGTTTCC | 160 |
| Chemokine (C-x-C motif) ligand 3 (*CXCL3*) | NM_001046513.2 | TCAACCCTGAAGCTCCCATG  AGTCCAGCACATCAAGTCCTT | 198 |
| Chemokine (C-C motif) receptor 7 (*CCR7*) | NM_001024930.3 | ATCATTGCTGTGGTCGTGGT  GAAAGGGTTGACACAGCAGC | 183 |
| Interleukin 13 receptor, alpha 1 (*IL13RA*) | NM_001206677.1 | CAGGTTGAGGCTGGAAGACA  CCCACCACTGCCATCTAAGT | 193 |
| ***Inflammatory cytokines*** | |  |  |
| Interleukin 1, alpha (*IL1-A*) | NM_174092.1 | AGAATGTGGTGATGGTGGCA  ACTTTGATTGAGGGCGTCGT | 224 |
| Interleukin 1, beta (*IL1-B*) | NM_174093.1 | GAAGAAAGGCCCGTCTTCCT  ACAGTGAAGTTCAGGCTGCA | 176 |
| Interleukin 6 (*IL6*) | NM_173923.2 | TGGTGATGACTTCTGCTTTCC  AGAGCTTCGGTTTTCTCTGG | 109 |
| Interleukin 10 (*IL10*) | NM_174088.1 | AGCTGTATCCACTTGCCAACC  TGGGTCAACAGTAAGCTGTGC | 119 |
| Tumor necrosis factor α (*TNFα*) | NM_173966.2 | CCACGTTGTAGCCGACATC  ACCACCAGCTGGTTGTCTTC | 108 |
| Transforming growth factor, beta 1 (*TGFβ1*) | NM_001166068.1 | CCTGGACACCAACTACTGCT  CCAGGACCTTGCTGTACTGT | 185 |
| ***Acute phase proteins / danger associated molecular pattern molecules*** | | | |
| Serum amyloid A3 (*SAA3*) | NM_001242573.1 | CACGGGCATCATTTTCTGCTT  GGGCAGCGTCATAGTTTCCA | 179 |
| Haptoglobin (*HP*) | NM_001040470.1 | AATGAACGATGGCTCCTCAC  TTGATGAGCCCAATGTCTACC | 176 |
| S100 calcium binding protein A9 (*S100A9*) | NM_001046328.1 | CTGGTGCAAAAAGAGCTGC  AGCATAATGAACTCCTCGAAGC | 128 |
| S100 calcium binding protein A12 (*S100A12*) | NM_174651.2 | TGGGGAGGCGCTGCTCTAGAC  TCGAAATGCCCCACCCGAACG | 135 |
| ***Antimicrobial peptides*** | |  |  |
| Lactoferrin (*LF*) | NM_180998.2 | CGAAGTGTGGATGGCAAGGAA  TTCAAGGTGGTCAAGTAGCGG | 215 |
| Lysozyme 1 K (*LYZ1*) | NM_001077829.1 | AAGAAACTTGGATTGGATGGC  ACTGCTTTTGGGGTTTTGC | 185 |
| Lactoperoxidase (*LPO*) | NM_173933.2 | TGGCTGTCAACCAAGAAGC  TGAGGCTCGAAAATCTCCC | 134 |
| Tracheal antimicrobial peptide (*TAP*) | NM_174776.1 | AGGAGTAGGAAATCCTGTAAGCTGTGT  AGCATTTTACTGCCCGCCCGA | 113 |
| Lingual antimicrobial peptide (*LAP*) | NM_203435.3 | AGAAATTCTCAAAGCTGCCG  CAGCATTTTACTTGGGCTCC | 107 |
| ***Apoptosis*** |  |  |  |
| Fas cell surface death receptor (*FAS*) | NM_174662.2 | CGGGATCTGGGTTCACTTGT  GGAGGACAAGGCTGACAACA | 180 |
| Tumor necrosis factor receptor superfamily, member 1A (*TNFRSF1A*) | NM_174674.2 | CGCCTCTGTCGTCTTAGCAT  GACTGGAACTTGGGGTGGAG | 170 |
| Tumor necrosis factor receptor 2 (*TNFR2*) | AF031589.1 | CCAGCAGCACGGACAAGA  CAATGCAGGTGACGTTGACC | 153 |
| Caspase 8 (*CASP8*) | NM_001045970.2 | TAGCATAGCACGGAAGCAGG  GCCAGTGAAGTAAGAGGTCAG | 295 |
| Caspase 3 (*CASP3*) | NM_001077840.1 | TCAGTCAGTCAGTTGGGCAC  GGGAGCATCTTCCACACACA | 164 |
| Caspase 1 (*CASP1*) | XM_002692921 | ACGTCTTGCCCTTATTATCTGC  GTACTGTCAGAGGTCCGATGC | 204 |
| BCL2-associated X protein (*BAX*) | NM_173894.1 | AGAGGATGATCGCAGCTGTG  GAAGTCCAATGTCCAGCCCA | 200 |
| Anti-apoptotic regulator Bcl-xL (*Bcl-xL*) | AF245487 | GGCATTCAGCGACCTGAC  CCATCCAAGTTGCGATCC | 203 |
| B-cell CLL/lymphoma 2 (*BCL-2*) | NM_001166486.1 | ATGTGTGTGGAGAGCGTCAA  GAGCAGTGCCTTCAGAGACA | 195 |
| ***Immunoglobulin receptors*** | |  |  |
| IgG Fc receptor (*FcRN*) | AF141017.1 | GAGCTGGCTCCTTGGATCTC  ATACCAGGATTCCCGGAGGT | 194 |
| Polymeric immunoglobulin receptor (*PIGR*) | NM_174143.1 | GACACCGTGGAGAGCAAAGA  GTGATTCGGAGCGTGATTGC | 192 |
| ***Scavenger Receptor*** |  |  |  |
| CD68 molecule (*CD68*) | NM_001045902.1 | GGCTCCAAGGAGGCAATAG  GAATGAGAGGAGCAAGTGGG | 201 |
| CD40 molecule (*CD40*) | NM_001105611.2 | TCGAAGGCCAACACTGTACC  GCCTTTTCTCTCGCAGCTTG | 197 |
| ***JAK-STAT signaling*** |  |  |  |
| Signal transducer and activator of transcription 2 (*STAT2*) | NM_001205689.1 | TCCTGCTGCGCTTTAGTGAA  GGATTCGCGGGTAGAGGAAG | 213 |
| ***Oxidative metabolism*** |  |  |  |
| Cytochrome P450, family 1, subfamily B, polypeptide 1 (*CYP1B1*) | NM_001192294.1 | GGACTTTGACCCAACCCGAT  CACTGGTGAGCAAGGATGGA | 159 |
| Cytochrome P450, family 1, subfamily A, polypeptide 1 (*CYP1A1*) | AF514290.1 | GGAGCCTAAAACCCACAGACA  CAGCACAACTTTGGAAGGGC | 177 |
| Nitric oxide synthase 2, inducible (*NOS2*) | NM_001076799.1 | CATTCGATGTCAGCGGCAAG  GCTGCGATTTGAGCCTCATG | 174 |
| ***MAPK signaling*** |  |  |  |
| FBJ murine osteosarcoma viral oncogene homolog (*FOS*) | NM_182786.2 | ACTGCTCGCGATCATGATGT  CCAGATCGGTGCAGTAGTCC | 173 |
| Mitogen-activated protein kinase 8 (*MAPK8*) | NM_001192974.1 | TGGAGGGGTAAAGGGCATTG  AGAAACGGCCAGGAAGTGTT | 156 |
| ***Others*** |  |  |  |
| Matrix metallopeptidase 1 (*MMP1*) | NM_174112 | TCTGGAGCAATGTCACACCC  CCTGCACCTGGTTGAAAAGC | 151 |
| Interferon regulatory factor 3 (*IRF3*) | NM_001029845.2 | GCTCAACTGACGGGAAGTGG  TTTGGGTTCCCATGGTCTGG | 128 |
| Myxovirus (influenza virus) resistance 1, interferon-inducible protein p78 (mouse) (*MX1*) | NM_173940.2 | AAGGCCACTATCCCCTGC  CTCGTACTTTGGTAAACAGTCGG | 277 |
| Myxovirus (influenza virus) resistance 2 (mouse) (*MX2*) | NM_173941.2 | CTTCAGAGACGCCTCAGTCG  TGAAGCAGCCAGGAATAGTG | 232 |
| Nucleotide-binding oligomerization domain containing 2 (*NOD2*) | NM_001002889.1 | CTGGCTCCGAGGAAACACTT  GTGCTCAGATGTCGTCCCAT | 158 |
| V-akt murine thymoma viral oncogene homolog 1 (*AKT1*) | NM_173986.2 | GATCACCGACTTCGGACTGT  CTTCTCGTGGTCCTGGTTGT | 202 |
| Wingless-type MMTV integration site family member 4 (*WNT4*) | XM_010826681.1 | CGGCCTTCACAGTGACTCTT  GGCCTAGGACAGTGTTTGCT | 150 |
| ***Reference genes*** |  |  |  |
| 18S ribosomal RNA gene (*18SrRNA*) | AF176811.1 | CGGGGAGGTAGTGACGAAA  CCGCTCCCAAGATCCAACTA | 195 |
| H3 histone, family 3A (*H3F3A*) | NM_001014389.2 | ACTTGCTACAAAAGCCGCTC  ACTTGCCTCCTGCAAAGCAC | 232 |
| Actin, gamma 1 (*ACTG1*) | NM_001033618 | AACTCCATCATGAAGTGTGAC  GATCCACATCTGCTGGAAGG | 234 |
| Glyceraldehyd-3-phosphate dehydrogenase (*GAPDH*) | NM_001034034.1 | GTCTTCACTACCATGGAGAAGG  TCATGGATGACCTTGGCCAG | 197 |
| Tyrosine 3-monoxygenase/tryptophan 5-monoxygenase activation protein, zeta polypeptide (*YWHAZ*) | NM_174814.2 | CAGGCTGAGCGATATGATGA  GACCCTCCAAGATGACCTAC | 141 |
| Cytokeratin 8 (*KRT8*) | NM_001033610 | TGGTGGAGGACTTCAAGACC  CGTGTCAGAAATCTGAGACTGC | 215 |
| Ubiquitine B (*UBB*) | NM_174133.2 | AGATCCAGGATAAGGAAGGCAT  GCTCCACCTCCAGGGTGAT | 426 |

^1^L = Length
