# Supplementary material for: Gene expression profiling in pbMEC – in search of molecular biomarkers to predict immunoglobulin production in bovine milk
Source: BMC Vet Res. 2017 Nov 29;13:369. doi: 10.1186/s12917-017-1293-z (PMC5707921; doi:10.1186/s12917-017-1293-z)
Supplement: Supplementary file 2 — Table S2. Fold changes in gene expression upon C. diff. Treatment - statistical evaluation of the treatment and time-effect with a paired t-test. High responder (n = 5), low responder (n = 4). (DOCX 40 kb) [file 12917_2017_1293_MOESM2_ESM.docx]

**Additional file 2**

***Table S2:*** Fold changes in gene expression upon *C. diff*. treatment - statistical evaluation of the treatment and time-effect with a paired t-test. High responder (n = 5), low responder (n = 4)

|  | | **time point** | | | | | | | | | | | | | | | | | | | | | | |
| --- | --- | --- | --- | --- | --- | --- | --- | --- | --- | --- | --- | --- | --- | --- | --- | --- | --- | --- | --- | --- | --- | --- | --- | --- |
| **genes** | | ***C. diff.* 6 h^1^** | | | | | ***C. diff.* 24 h^1^** | | | | | | | | | ***C. diff.* 72 h^1^** | | | | | | | | |
|  | | **High^2^** | | **Low^3^** | | | **High^2^** | | | | | **Low^3^** | | | | | **High^2^** | | | | **Low^3^** | | | |
| ***TLR pathway*** | | | | | | | | | | | | | | | | | | | | | | | | |
| *TLR2* | Fold | 1.51 |  | 1.14 |  | | 1.29 | |  | | 1.34 | | |  | | 1.40 | | |  | 1.08 | | |  | |
|  | SEM | 0.37 |  | 0.35 |  | | 0.21 | |  | | 0.24 | | |  | | 0.22 | | |  | 0.19 | | |  | |
| *TLR4* | Fold | 1.24 |  | 1.10 |  | | 1.03 | |  | | 0.87 | | | + | | 0.87 | | | * | 1.02 | | |  | |
|  | SEM | 0.16 |  | 0.06 |  | | 0.09 | |  | | 0.08 | | |  | | 0.08 | | |  | 0.13 | | |  | |
| *LY96* | Fold | 1.05 | A | 0.91 | +a | | 1.19 | | +B | | 1.03 | | | b | | 0.89 | | | +B | 1.01 | | | b | |
|  | SEM | 0.11 |  | 0.05 |  | | 0.08 | |  | | 0.09 | | |  | | 0.07 | | |  | 0.13 | | |  | |
| *LBP* | Fold | 1.65 |  | 1.50 | a | | 0.96 | | + | | 1.57 | | | ab | | 0.78 | | | * | 0.81 | | | +b | |
|  | SEM | 0.60 |  | 0.46 |  | | 0.25 | |  | | 0.59 | | |  | | 0.20 | | |  | 0.12 | | |  | |
| *CD14* | Fold | 1.06 | A | 0.99 | a | | 1.70 | | +B | | 0.78 | | | ab | | 1.64 | | | B | 1.05 | | | b | |
|  | SEM | 0.29 |  | 0.10 |  | | 0.34 | |  | | 0.14 | | |  | | 0.36 | | |  | 0.10 | | |  | |
| *MYD88* | Fold | 1.03 | A | 1.28 | *a | | 1.18 | | +A | | 1.04 | | | b | | 0.95 | | | B | 0.91 | | | *c | |
|  | SEM | 0.09 |  | 0.09 |  | | 0.08 | |  | | 0.05 | | |  | | 0.09 | | |  | 0.08 | | |  | |
| *TIRAP* | Fold | 1.53 | *** | 1.19 | *a | | 1.25 | | ** | | 1.04 | | | a | | 1.15 | | |  | 0.99 | | | b | |
|  | SEM | 0.12 |  | 0.07 |  | | 0.07 | |  | | 0.08 | | |  | | 0.09 | | |  | 0.08 | | |  | |
| *TRAF6* | Fold | 1.30 | **A | 1.22 | ***a | | 1.14 | | AB | | 0.91 | | | ab | | 1.00 | | | B | 0.91 | | | b | |
|  | SEM | 0.07 |  | 0.05 |  | | 0.11 | |  | | 0.07 | | |  | | 0.09 | | |  | 0.08 | | |  | |
| *IRAK4* | Fold | 1.11 | A | 1.00 | a | | 1.12 | | B | | 1.13 | | | ab | | 1.38 | | | A | 1.17 | | | b | |
|  | SEM | 0.16 |  | 0.11 |  | | 0.10 | |  | | 0.21 | | |  | | 0.20 | | |  | 0.17 | | |  | |
| *IRAK1* | Fold | 1.06 | A | 1.00 |  | | 1.14 | | *B | | 1.13 | | |  | | 0.95­ | | | B | 1.17 | | |  | |
|  | SEM | 0.08 |  | 0.11 |  | | 0.05 | |  | | 0.21 | | |  | | 0.07 | | |  | 0.17 | | |  | |
| *RELA* | Fold | 1.22 | *A | 1.27 | *a | | 1.11 | | A | | 0.97­ | | | b | | 1.23­ | | | B | 0.80 | | | +c | |
|  | SEM | 0.08 |  | 0.10 |  | | 0.06 | |  | | 0.06 | | |  | | 0.32 | | |  | 0.12 | | |  | |
| ***Complement system*** | | | | | | | | | | | | | | | | | | | | | | | | |
| *C3* | Fold | 2.13 | A | 1.36 | a | | 0.42 | | ***A | | 0.88 | | | a | | 0.65 | | | **B | 0.90 | | | b | |
|  | SEM | 0.70 |  | 0.51 |  | | 0.09 | |  | | 0.26 | | |  | | 0.15 | | |  | 0.19 | | |  | |
| ***Chemokines*** | | | | | | | | | | | | | | | | | | | | | | | | |
| *CCL5* | Fold | 1.47 |  | 0.72 | | **a | | 1.16 | |  | | | 0.94 | | a | | | 0.74 | * | | | 0.99 | | b |
|  | SEM | 0.27 |  | 0.11 | |  | | 0.16 | |  | | | 0.17 | |  | | | 0.21 |  | | | 0.12 | |  |
| *CCL20* | Fold | 1.14 | A | 1.30 | | a | | 0.53 | | ***A | | | 0.52 | | **a | | | 2.08 | **B | | | 0.94 | | b |
|  | SEM | 0.21 |  | 0.21 | |  | | 0.06 | |  | | | 0.10 | |  | | | 0.32 |  | | | 0.12 | |  |
| *CXCL5* | Fold | 1.30 | A | 1.10 | | a | | 0.97­ | | *B | | | 1.12 | | b | | | 1.21 | A | | | 1.14 | | c |
|  | SEM | 0.20 |  | 0.10 | |  | | 0.12 | |  | | | 0.16 | |  | | | 0.18 |  | | | 0.32 | |  |
| *CXCL8* | Fold | 2.01 | ***A | 1.31 | |  | | 0.84 | | *A | | | 0.84 | | + | | | 1.41 | B | | | 0.98 | |  |
|  | SEM | 0.22 |  | 0.16 | |  | | 0.10 | |  | | | 0.12 | |  | | | 0.27 |  | | | 0.13 | |  |
| *CXCL3* | Fold | 1.56 | *A | 1.5 | | **a | | 1.14 | | B | | | 1.17 | | b | | | 1.23 | B | | | 1.19 | | b |
|  | SEM | 0.16 |  | 0.16 | |  | | 0.10 | |  | | | 0.14 | |  | | | 0.17 |  | | | 0.30 | |  |
| *CCR7* | Fold | 1.43 | A | 0.88 | | +a | | 2.35 | | B | | | 2.85 | | **b | | | 0.66 | **A | | | 0.95 | | ab |
|  | SEM | 0.37 |  | 0.23 | |  | | 0.74 | |  | | | 0.74 | |  | | | 0.14 |  | | | 0.15 | |  |
| *IL13RA* | Fold | 1.12 | A | 1.09 | | a | | 1.20 | | +B | | | 1.02 | | ab | | | 1.12 | B | | | 0.89 | | b |
|  | SEM | 0.07 |  | 0.08 | |  | | 0.09 | |  | | | 0.12 | |  | | | 0.08 |  | | | 0.08 | |  |

|  | | **time point** | | | | | | | | | | | | | | | | |
| --- | --- | --- | --- | --- | --- | --- | --- | --- | --- | --- | --- | --- | --- | --- | --- | --- | --- | --- |
| genes | | ***C. diff.* 6 h^1^** | | | | | | | | ***C. diff.* 24 h^1^** | | | | | ***C. diff.* 72 h^1^** | | | |
|  | | **High^2^** | | | | **Low^3^** | | | | **High^2^** | | | **Low^3^** | | **High^2^** | | **Low^3^** | |
| ***Inflammatory cytokines*** | | | | | | | | | | | | | | | | | | |
| *IL1-A* | Fold | 1.18 | |  | | 0.97 | | a | | 0.76 | | ** | 0.71 | **ab | 2.51 | *** | 1.04 | b |
|  | SEM | 0.17 | |  | | 0.08 | |  | | 0.06 | |  | 0.07 |  | 0.54 |  | 0.12 |  |
| *IL6* | Fold | 0.43 | | **A | | 0.70 | | **a | | 1.52 | | ***B | 1.27 | *b | 2.00 | A | 1.01 | a |
|  | SEM | 0.07 | |  | | 0.08 | |  | | 0.12 | |  | 0.10 |  | 0.63 |  | 0.13 |  |
| *TNFα* | Fold | 0.91 | |  | | 1.31 | |  | | 0.86 | | + | 0.80 | * | 2.25 |  | 1.14 |  |
|  | SEM | 0.16 | |  | | 0.22 | |  | | 0.14 | |  | 0.12 |  | 0.57 |  | 0.24 |  |
| *TGFβ1* | Fold | 0.94 | |  | | 1.18 | | * | | 0.85 | | * | 0.93 |  | 0.76 | ** | 0.97 |  |
|  | SEM | 0.06 | |  | | 0.07 | |  | | 0.08 | |  | 0.08 |  | 0.09 |  | 0.10 |  |
| ***Acute phase proteins / danger associated molecular pattern molecules*** | | | | | | | | | | | | | | | | | | |
| *SAA3* | Fold | 2.77 | | AB | | 1.26 | | a | | 0.57 | | ***A | 1.52 | ab | 1.08 | B | 1.25 | b |
|  | SEM | 1.06 | |  | | 0.26 | |  | | 0.22 | |  | 0.68 |  | 0.28 |  | 0.43 |  |
| *S100A9* | Fold | 1.16 | | A | | 1.25 | | a | | 1.70 | | ***B | 1.52 | **b | 2.26 | +C | 0.99 | c |
|  | SEM | 0.11 | |  | | 0.16 | |  | | 0.19 | |  | 0.16 |  | 0.56 |  | 0.16 |  |
| *S100A12* | Fold | 1.26 | | A | | 0.87 | | + | | 2.20 | | ***B | 1.71 | + | 2.02 | *B | 0.95 |  |
|  | SEM | 0.22 | |  | | 0.07 | |  | | 0.29 | |  | 0.24 |  | 0.56 |  | 0.19 |  |
| ***Antimicrobial peptides*** | | | | | | | | | | | | | | | | | | |
| *LF* | Fold | 1.53 | A | | 1.16 | | a | | 0.62 | | ***B | | 1.21 | b | 0.69 | *B | 0.78 | *B |
|  | SEM | 0.42 |  | | 0.12 | |  | | 0.09 | |  | | 0.37 |  | 0.15 |  | 0.13 |  |
| *LYZ1* | Fold | 1.90 | A | | 0.97 | | a | | 0.71 | | **B | | 1.10 | a | 3.04 | ***C | 1.65 | *b |
|  | SEM | 0.39 |  | | 0.10 | |  | | 0.09 | |  | | 0.17 |  | 0.72 |  | 0.36 |  |
| *LPO* | Fold | 1.16 |  | | 1.06 | |  | | 1.38 | |  | | 1.04 |  | 1.58 | * | 1.02 |  |
|  | SEM | 0.15 |  | | 0.10 | |  | | 0.24 | |  | | 0.19 |  | 0.23 |  | 0.15 |  |
| ***Apoptosis*** | | | | | | | | | | | | | | | | | | |
| *FAS* | Fold | 1.21 | *A | | 1.21 | | **a | | 1.82 | | *B | | 0.93 | a | 1.42 | *A | 0.89 | +b |
|  | SEM | 0.08 |  | | 0.07 | |  | | 0.40 | |  | | 0.09 |  | 0.16 |  | 0.07 |  |
| *TNFRSF1A* | Fold | 1.10 |  | | 1.35 | | *a | | 1.08 | |  | | 0.91 | ab | 1.01 |  | 0.97 | b |
|  | SEM | 0.09 |  | | 0.15 | |  | | 0.09 | |  | | 0.09 |  | 0.12 |  | 0.12 |  |
| *TNFR2* | Fold | 1.17 |  | | 1.10 | |  | | 0.91 | |  | | 0.92 |  | 0.84 | * | 1.06 |  |
|  | SEM | 0.12 |  | | 0.14 | |  | | 0.09 | |  | | 0.12 |  | 0.13 |  | 0.19 |  |
| *CASP8* | Fold | 1.14 | A | | 1.09 | | +a | | 1.24 | | B | | 0.93 | b | 1.07 | C | 1.02 | b |
|  | SEM | 0.11 |  | | 0.05 | |  | | 0.12 | |  | | 0.08 |  | 0.12 |  | 0.11 |  |
| *CASP3* | Fold | 1.27 | A | | 1.08 | | a | | 0.99 | | B | | 0.89 | b | 0.91 | +B | 0.96 | b |
|  | SEM | 0.13 |  | | 0.10 | |  | | 0.07 | |  | | 0.09 |  | 0.14 |  | 0.11 |  |
| *BAX* | Fold | 1.15 | +A | | 1.00 | | a | | 1.05 | | A | | 1.00 | a | 1.56 | B | 1.33 | b |
|  | SEM | 0.10 |  | | 0.04 | |  | | 0.05 | |  | | 0.05 |  | 0.30 |  | 0.25 |  |
| *BCL-xL* | Fold | 0.87 |  | | 1.01 | |  | | 1.04 | |  | | 0.92 |  | 1.18 |  | 1.07 |  |
|  | SEM | 0.07 |  | | 0.07 | |  | | 0.07 | |  | | 0.08 |  | 0.10 |  | 0.13 |  |
| *BCL-2* | Fold | 0.58 | ***A | | 1.17 | | a | | 0.77 | | *AB | | 0.79 | *b | 0.83 | *B | 1.11 | b |
|  | SEM | 0.06 |  | | 0.18 | |  | | 0.11 | |  | | 0.07 |  | 0.12 |  | 0.17 |  |
| ***Immunoglobulin receptors*** | | | | | | | | | | | | | | | | | | |
| *FcRN* | Fold | 1.37 |  | | 0.96 | | a | | 1.09 | |  | | 1.05 | b | 0.81 | * | 1.04 | ab |
|  | SEM | 0.39 |  | | 0.19 | |  | | 0.13 | |  | | 0.12 |  | 0.09 |  | 0.17 |  |
| *PIGR* | Fold | 0.89 | A | | 0.90 | |  | | 0.83 | | +B | | 0.70 | ** | 0.56 | **AB | 0.62 | * |
|  | SEM | 0.15 |  | | 0.16 | |  | | 0.12 | |  | | 0.09 |  | 0.15 |  | 0.14 |  |

|  | | **time point** | | | | | | | | | | | | | | | | | | | | | | | | | | | | | | |
| --- | --- | --- | --- | --- | --- | --- | --- | --- | --- | --- | --- | --- | --- | --- | --- | --- | --- | --- | --- | --- | --- | --- | --- | --- | --- | --- | --- | --- | --- | --- | --- | --- |
| genes | | ***C. diff.* 6 h^1^** | | | | | | | | | | ***C. diff.* 24 h^1^** | | | | | | | | | | ***C. diff.* 72 h^1^** | | | | | | | | | | |
|  | | **High^2^** | | | | | **Low^3^** | | | | | **High^2^** | | | | | **Low^3^** | | | | | **High^2^** | | | | | **Low^3^** | | | | | |
| ***Scavenger Receptor*** | | | | | | | | | | | | | | | | | | | | | | | | | | | | | | | | |
| *CD68* | Fold^4^ | | | 1.84 | | **A | | | 1.38 | | a | | | 1.38 | | B | | | 0.92 | | b | | | 3.87 | | +C | | | 1.24 | | c | |
|  | SEM | | | 0.29 | |  | | | 0.30 | |  | | | 0.19 | |  | | | 0.12 | |  | | | 2.31 | |  | | | 0.34 | |  | |
| *CD40* | Fold | | | 1.12 | |  | | | 1.08 | | a | | | 0.97 | |  | | | 0.88 | | b | | | 1.27 | |  | | | 1.05 | | ab | |
|  | SEM | | | 0.08 | |  | | | 0.05 | |  | | | 0.04 | |  | | | 0.10 | |  | | | 0.14 | |  | | | 0.14 | |  | |
| ***JAK-STAT signaling*** | | | | | | | | | | | | | | | | | | | | | | | | | | | | | | | | |
| *STAT2* | Fold | | | 1.20 | |  | | | 1.15 | | * | | | 1.21 | |  | | | 0.94 | |  | | | 1.06 | |  | | | 1.08 | |  | |
|  | SEM | | | 0.16 | |  | | | 0.06 | |  | | | 0.11 | |  | | | 0.11 | |  | | | 0.12 | |  | | | 0.13 | |  | |
| ***Oxidative metabolism*** | | | | | | | | | | | | | | | | | | | | | | | | | | | | | | | | |
| *CYP1B1* | Fold | | | 1.08 | | A | | | 1.15 | | a | | | 1.06 | | B | | | 1.17 | | b | | | 2.09 | | C | | | 1.25 | | c | |
|  | SEM | | | 0.11 | |  | | | 0.08 | |  | | | 0.09 | |  | | | 0.12 | |  | | | 0.51 | |  | | | 0.21 | |  | |
| *NOS2* | Fold | | | 1.55 | | A | | | 1.19 | | a | | | 1.09 | | AB | | | 1.66 | | a | | | 1.33 | | B | | | 1.28 | | b | |
|  | SEM | | | 0.30 | |  | | | 0.20 | |  | | | 0.12 | |  | | | 0.38 | |  | | | 0.17 | |  | | | 0.21 | |  | |
| **MAPK signaling** | | | | | | | | | | | | | | | | | | | | | | | | | | | | | | | | |
| *Fos* | Fold | | 1.28 | | ***A | | | 1.25 | | | * | | | 0.91 | | *B | | | 0.99 | |  | | | 1.01 | | A | | | 1.00 | |  | |
|  | SEM | | 0.06 | | |  | | | 0.07 | |  | | | 0.05 | |  | | | 0.09 | |  | | | 0.12 | |  | | | 0.08 | |  | |
| *MAPK8* | Fold | | 1.34 | | | A | | | 0.98 | | a | | | 1.32 | | +B | | | 1.01 | | b | | | 0.74 | | *C | | | 0.92 | | a | |
|  | SEM | | 0.23 | | |  | | | 0.13 | |  | | | 0.14 | |  | | | 0.10 | |  | | | 0.11 | |  | | | 0.11 | |  | |
| ***Others*** | | | | | | | | | | | | | | | | | | | | | | | | | | | | | | | | |
| *MMP1* | Fold | | 1.56 | | ***A | | | 1.19 | | **a | | | 2.22 | | ***B | | | 1.06 | | ab | | | 2.42 | | **B | | | 0.95 | | b | |  |
|  | SEM | | 0.11 | |  | | | 0.06 | |  | | | 0.42 | |  | | | 0.10 | |  | | | 0.50 | |  | | | 0.07 | |  | |  |
| *IRF3* | Fold | | 1.31 | | A | | | 1.08 | | a | | | 1.03 | | B | | | 0.95 | | a | | | 1.02 | | B | | | 1.18 | | b | |  |
|  | SEM | | 0.31 | |  | | | 0.13 | |  | | | 0.10 | |  | | | 0.09 | |  | | | 0.18 | |  | | | 0.22 | |  | |  |
| *MX1* | Fold | | 1.13 | | A | | | 1.06 | | a | | | 0.73 | | B | | | 1.30 | | b | | | 1.06 | | C | | | 0.90 | | b | |  |
|  | SEM | | 0.18 | |  | | | 0.22 | |  | | | 0.11 | |  | | | 0.61 | |  | | | 0.16 | |  | | | 0.19 | |  | |  |
| *MX2* | Fold | | 0.99 | | A | | | 1.32 | | a | | | 0.83 | | +B | | | 1.27 | | b | | | 1.19 | | C | | | 0.54 | | **b | |  |
|  | SEM | | 0.20 | |  | | | 0.42 | |  | | | 0.14 | |  | | | 0.50 | |  | | | 0.16 | |  | | | 0.10 | |  | |  |
| *NOD2* | Fold | | 0.94 | | +A | | | 0.97 | | a | | | 1.04 | | B | | | 0.94 | | b | | | 1.15 | | C | | | 0.72 | | *c | |  |
|  | SEM | | 0.24 | |  | | | 0.11 | |  | | | 0.28 | |  | | | 0.11 | |  | | | 0.21 | |  | | | 0.14 | |  | |  |
| *AKT1* | Fold | | 1.09 | |  | | | 1.12 | | a | | | 0.83 | | ** | | | 0.91 | | +a | | | 0.97 | |  | | | 0.96 | | b | |  |
|  | SEM | | 0.08 | |  | | | 0.11 | |  | | | 0.06 | |  | | | 0.06 | |  | | | 0.16 | |  | | | 0.13 | |  | |  |
| *WNT4* | Fold | | 0.92 | | A | | | 1.78 | |  | | | 1.10 | | B | | | 1.22 | |  | | | 0.98 | | B | | | 0.63 | | ** | |  |
|  | SEM | | 0.17 | |  | | | 0.55 | |  | | | 0.17 | |  | | | 0.25 | |  | | | 0.19 | |  | | | 0.10 | |  | |  |
|  |  | |  | |  | | |  | |  | | |  | |  | | |  | |  | | |  | |  | | |  | |  | |  |

^1^Treatment time with *C. diff.* in hours

^2^High = High responder animals (n=5)

^3^Low = Low responder animals (n=4)

^4^Fold = Fold Change [2^^(-ΔΔCq)^]

* p ≤ 0.05, ** p≤ 0.01, ***p≤ 0.001 between treatment and control

+ Distinct changes (0.01 ≤ p < 0.05) between treatment and control

Upper case letter: significant changes between treatment time-points high responder animals

Lower case letter: significant changes between treatment time-points low responder animals
